# Guida pratica alla prevenzione e gestione dell’infezione da COVID-19 nelle persone con diabete

**DOI:** 10.1007/s40619-020-00767-3

**Published:** 2020-10-23

**Authors:** Matteo Apicella, Maria Cristina Campopiano, Michele Mantuano, Laura Mazoni, Stefano Del Prato

**Affiliations:** Dipartimento di Medicina Clinica e Sperimentale, Università di Pisa e Azienda Ospedaliero Universitaria Pisana, Pisa, Italia

**Keywords:** Diabete mellito, COVID-19, Comorbilità, Prognosi, Farmaci anti-diabetici, Terapia

## Abstract

La pandemia di COVID-19 rappresenta un’enorme sfida per il sistema sanitario nazionale. Sulla base dei dati ad oggi disponibili è emerso che le persone con diabete mellito presentano un maggior rischio di complicanze e morte per COVID-19. Pertanto, adottare misure preventive di igiene e di distanziamento sociale è cruciale, a maggior ragione in questa categoria di soggetti. A sostegno dei pazienti con diabete sono state intraprese molteplici iniziative al fine di garantire la continuità assistenziale, tra cui la proroga della validità dei piani terapeutici per i farmaci ipoglicemizzanti, le procedure per il rinnovo della patente di guida e l’attivazione di servizi di telemedicina. In caso di infezione da COVID-19 e sintomi lievi è possibile una gestione domiciliare della persona con diabete, raccomandando un attento monitoraggio glicemico. Il paziente diabetico che necessita di ospedalizzazione richiede una gestione multidisciplinare che includa il diabetologo, con l’obiettivo di mantenere un adeguato controllo glicemico in assenza di ipoglicemie. Le persone con diabete rappresentano un gruppo vulnerabile per il quale devono essere poste in atto strategie specifiche sia in termini di prevenzione che di trattamento.

**Materiale elettronico supplementare:**

La versione elettronica di questo articolo (10.1007/s40619-020-00767-3) contiene materiale supplementare, disponibile per gli utenti autorizzati.

## Introduzione

Nel dicembre 2019 vennero identificati a Wuhan, in Cina, alcuni casi di polmonite interstiziale atipica causati da un nuovo coronavirus in grado di infettare l’uomo, denominato SARS-CoV-2. La malattia respiratoria acuta da SARS-CoV-2 o COVID-19 si è quindi rapidamente diffusa dall’Asia al resto del mondo, tanto che l’Organizzazione Mondiale della Sanità (OMS) l’11 marzo 2020 ha dichiarato lo stato di pandemia.

Numerose società scientifiche e gruppi di ricerca hanno tentato di definire le categorie maggiormente a rischio di infezione e, fra queste, grande attenzione è stata riservata ai pazienti con diabete mellito, considerata l’elevata prevalenza di tale condizione. È noto come la popolazione diabetica sia maggiormente suscettibile alle infezioni, incluse quelle polmonari. Non a caso, le persone con diabete rientrano tra le categorie per le quali è raccomandata la vaccinazione per l’influenza stagionale. Un aumento del rischio di infezione era stato già registrato per SARS [1], H1N1 [2] e MERS [3], mentre per COVID-19 i dati sono al momento parziali e non univoci. In un recente lavoro italiano che ha considerato 146 pazienti ospedalizzati per COVID-19 con età media di 65,3 anni, la prevalenza di diabete mellito era dell’8,9% contro una percentuale dell’11% nella popolazione della stessa area geografica e fascia di età. Quando gli stessi autori hanno valutato la prevalenza di diabete tra i pazienti deceduti per COVID-19, questa percentuale saliva al 35,5%. Sulla base di tali dati, si concludeva che il diabete non aumenterebbe il rischio di contrarre l’infezione ma potrebbe condizionarne in senso negativo l’evoluzione e l’esito [4]. Nella stessa direzione va la recente meta-analisi di Bo Li et al. che ha preso in considerazione sei studi cinesi, per un totale di 1.527 soggetti con COVID-19. Anche in questo caso non si rincontrava un’aumentata suscettibilità all’infezione per i pazienti con ipertensione arteriosa e diabete mellito [5].

Guo W e collaboratori hanno osservato una mortalità del 16% per COVID-19 in un gruppo di 24 pazienti con diabete mellito e nessun’altra co-morbilità, mentre nessun decesso veniva registrato in 26 controlli non diabetici. Gli stessi autori suggeriscono che l’infezione possa esordire con sintomi più lievi e sfumati nel soggetto diabetico, rallentandone l’iter diagnostico e ritardando l’intervento terapeutico [6, 7]. Altri aspetti restano comunque da chiarire: fra gli altri, se il rischio sia legato al grado di compenso glicemico, alla malattia diabetica in senso lato o alle co-morbilità spesso presenti, soprattutto nei pazienti con diabete mellito tipo 2. Per quanto riguarda quest’ultima possibilità, esistono già evidenze di come la presenza di due o più patologie concomitanti peggiori la prognosi dei soggetti con infezione da COVID-19 [5, 8, 9].

Alla luce di questa breve disanima, è evidente come le persone con diabete debbano essere adeguatamente informate del rischio infettivo e edotte sui comportamenti da adottare per ridurre il rischio di contagio. Nel contempo, la classe medica deve essere in grado di continuare a offrire la necessaria assistenza a tutte le persone con malattie croniche, ivi incluse quelle con diabete, nonostante le difficoltà ad accedere alle prestazioni ambulatoriali.

## Regole di igiene e raccomandazioni generali per la persona con diabete

Arginare la diffusione del virus rimane un obiettivo primario e inderogabile. Per questo motivo, le normali attività ambulatoriali sono state limitate ai casi urgenti e non procrastinabili. Questo a salvaguardia delle persone più a rischio, come la persona con diabete, ma anche dello stesso personale sanitario. Le regole emanate a livello nazionale ancor più trovano indicazione nelle persone con diabete. In quest’ottica devono essere lette le limitazioni imposte a partire dal 9 marzo u.s. relative all’uscire da casa, incluso l’accesso ai Servizi di Diabetologia. Proprio per andare incontro alle esigenze della persona con diabete è utile ricordare che i piani terapeutici per i farmaci anti-iperglicemizzanti sottoposti a controllo (DPP4-inibitori, SGLT2-inibitori, agonisti del GLP1, nuovi analoghi dell’insulina basale) e le richieste di presidi in scadenza da febbraio a maggio 2020 sono state automaticamente prorogate di 90 giorni con la possibilità di un’ulteriore proroga allo scadere di quella data. Analogamente, è stata prolungata la validità della patente di guida fino alla fine di agosto.

Al fine di garantire un’adeguata assistenza, i Servizi di Diabetologia hanno attivato procedure di telemedicina e teleconsulto. Questa attività, formalmente riconosciuta e identificata mediante apposito codice di nomenclatore, ha permesso di ricontattare la stragrande maggioranza delle persone già in possesso di appuntamento ambulatoriale, così come ha consentito di creare un punto di contatto con lo specialista di diabetologia sia per la persona con diabete che per il Medico di Medicina Generale. Questo approccio rappresenta, ovviamente, una novità e un passo nel futuro della gestione delle patologie endocrinologiche e metaboliche. Riecheggiando le parole di Trimarchi [10], d’ora in avanti l’endocrinologo e il diabetologo dovranno imparare a gestire anche a distanza diagnosi e terapie senza perdere di vista aspettative e preoccupazioni delle persone.

Direttive gestionali dettagliate sono state congiuntamente fornite dalla Società Italiana di Diabetologia (SID) e dall’Associazione Medici Diabetologi (AMD) in collaborazione con la Società Italiana di Endocrinologia (SIE). Queste Società, con la Società Italiana di Endocrinologia e Diabetologia Pediatrica (SIEDP), hanno anche attivato sulla piattaforma Facebook il servizio “Un’ora con AMD, SID, SIEDP” dove lo specialista offre informazioni e aggiornamenti alle persone con diabete [11].

A parte queste specifiche iniziative, valgono ovviamente tutte le regole generali di igiene personale cui le persone con diabete devono attenersi con il massino scrupolo e cioè:

- lavare spesso le mani accuratamente per almeno 60 secondi con acqua e sapone o, se non disponibile, con un disinfettante a base di alcool con concentrazione alcoolica $\geq70\%$. Tale pratica è ancora più importante nella persona con diabete che usa spesso le mani per la gestione della propria malattia: controllo della glicemia con gli aghi pungi-dito, manipolazione dei farmaci antidiabetici e, in particolare, i dispositivi per la somministrazione dell’insulina;
- mantenimento, nei contatti sociali, di una distanza sociale di almeno un metro e uso della mascherina secondo indicazioni;
- evitare abbracci e strette di mano;
- avere cura di starnutire o tossire coprendo naso e bocca con un fazzoletto usa e getta oppure nell’incavo del gomito, evitando il contatto delle mani con le secrezioni respiratorie;
- evitare di toccarsi occhi, naso e bocca con le mani;
- evitare l’uso promiscuo di stoviglie e bicchieri;
- pulire le superfici con disinfettanti a base di cloro o alcool;
- usare la mascherina soprattutto in presenza di sintomi compatibili con COVID19 (febbre, tosse, congestione nasale, dispnea) o se si presta assistenza a persone malate. Prima di indossare la mascherina è consigliato il lavaggio delle mani con acqua e sapone o con soluzione alcoolica; nell’indossare la mascherina è importante evitare di toccarla, prendendola dall’elastico e avendo cura di coprire bocca e naso con essa, facendola aderire bene al volto. Nel togliere la mascherina (sempre prendendola dall’elastico) si deve aver cura di non toccare la sua parte anteriore, gettandola in un sacchetto chiuso e lavandosi le mani;
- non assumere farmaci antivirali e antibiotici, se non prescritti dal medico;
- in caso di comparsa di febbre, tosse o dispnea non recarsi al Pronto Soccorso o presso gli studi medici, ma contattare il Medico di famiglia, il pediatra, la guardia medica, il Servizio di Diabetologia o l’apposito numero verde regionale.

Questa situazione emergenziale può esercitare un impatto sulla gestione e controllo della glicemia della persona con diabete. Infatti, le misure di contenimento adottate per contrastare la diffusione del virus espongono al rischio di perdita di aderenza alle regole di igiene di vita. Alla persona con diabete va quindi ricordata la necessità di mantenere un equilibrato stile di vita ai fini anche della salvaguardia della salute psicofisica. A questo riguardo, il Ministero della Salute ha pubblicato sull’apposito sito una pagina intera dedicata al tema degli stili di vita ai tempi della COVID-19 nel quale vengono fornite semplici e pratiche indicazioni da seguire [12].

Mantenere uno stile di attivo durante la pandemia COVID-19 può essere una sfida. Le opportunità di essere fisicamente attivi sembrano essere più limitate e proprio per questo è ancora più importante pianificare l’attività fisica giornaliera limitando il tempo trascorso seduti. Anche tra le mura domestiche una sufficiente attività fisica può essere garantita con semplici accorgimenti quali salire e scendere più volte le scale; partecipare a lezioni di ginnastica online gratuite; utilizzare attrezzi da ginnastica come cyclette, tapis roulant, elastici o piccoli pesi, se disponibili; correre e saltare sul posto, ecc.

Non ultimo, bisogna ricordare alle persone con diabete di non procrastinare il contatto telefonico preventivo con il Medico di Medicina Generale e/o con il Diabetologo di riferimento in caso di segni o sintomi riferibili a condizioni di urgenza sia legate alle complicanze della malattia (scompensi acuti, riscontro di chetoni nelle urine, lesioni e ulcerazioni alle estremità, disturbi visivi importanti e subitanei, ecc.), sia quelle per cui è dimostrato un aumentato rischio (infarto, ictus, arteriopatie obliteranti degli arti inferiori, ecc.). Sarà cura del personale sanitario valutare la necessità di raccomandare l’eventuale accesso al Pronto Soccorso o alle strutture cliniche di competenza.

## Gestione della persona con diabete e infezione da COVID-19 a domicilio

I pazienti diabetici con infezione da COVID-19 con una sintomatologia tale da non richiedere il ricovero ospedaliero devono essere attentamente gestiti a domicilio, dove devono rimanere isolati per almeno 14 giorni e, comunque, fintanto che i sintomi si risolvano, facendo regolare uso di mascherina chirurgica. Anche se la componente polmonare è tale da non richiedere la sorveglianza in ambito ospedaliero, va sempre ricordato che la patologia intercorrente può esporre al rischio di un rapido scompenso glicemico. In queste situazioni valgono quelle che gli anglosassoni definiscono le *Sick Day Rules*. Queste regole prevedono un’adeguata idratazione (che potrebbe diventare ancora più importante in caso di sintomatologia gastro-intestinale), un più stretto monitoraggio della glicemia e l’adattamento della terapia soprattutto nel caso della persona con diabete insulino-trattato. L’alimentazione deve essere bilanciata, eventualmente supportata con integratori alimentari in caso di riduzione dell’appetito o di disturbi gastrointestinali. In caso di febbre e iperglicemia è necessario verificare la presenza di chetoni nelle urine o nel sangue mediante gli appositi dispositivi. Per valori di chetonuria $>2+$ o di chetonemia $>3~\mbox{mmol/l}$, il centro diabetologico o il medico di riferimento deve essere immediatamente contattato [13]. Eventuali modifiche della terapia antidiabetica dovranno essere valutate dal personale sanitario sulla base delle caratteristiche cliniche del paziente, dei valori glicemici registrati e dal tipo di trattamento farmacologico in atto. Ovviamente, può essere indicato l’uso di terapia sintomatica, ad esempio paracetamolo, in caso di febbre. L’uso di antinfiammatori non-steroidei (FANS), incluso l’ibuprofene, non è indicato dato un possibile effetto di soppressione immunitaria che potrebbe ritardare la risoluzione del quadro, oltre che favorire una ritenzione di sodio e di liquidi che potrebbero peggiorare il quadro polmonare.

## Gestione della persona con diabete ospedalizzata per infezione da COVID-19

Particolare attenzione dovrà essere riservata ai pazienti diabetici ospedalizzati per COVID-19. Anche in ospedale, un buon controllo della glicemia parte dalla dieta e dalla regolare assunzione della terapia anti-iperglicemizzante. In uno studio condotto su un gruppo di diabetici affetti da COVID-19 nel distretto di Wuhan, è emerso che il 69% dei pazienti presentava glicemie non al target; l’assenza di un servizio dietetico dedicato figurava tra le possibili cause del mancato controllo metabolico [14].

Per quanto non esistano evidenze specifiche nel caso dell’infezione da COVID-19, è comunque auspicabile che un adeguato controllo venga raggiunto in assenza di ipoglicemia, perché sia glicemie elevate che glicemie troppo basse sono state correlate a esiti peggiori in caso di patologie acute intercorrenti nella persona con diabete [15–17].

Altro punto di primaria importanza riguarda la scelta della terapia anti-iperglicemizzante più appropriata. A tale proposito, l’Associazione Medici Diabetologi consiglia la sospensione degli antidiabetici non insulinici nel paziente ospedalizzato per COVID19, preferendo il passaggio al trattamento con insulina [18]. La terapia insulinica, in questi casi, deve avvenire secondo lo schema *basal-bolus* mentre lo schema *sliding scale*, ovvero l’esclusivo impiego di insulina rapida *al bisogno* o con dosi modulate sui valori estemporanei della glicemia è da evitare, in quanto è gravata da un maggior rischio di ipoglicemia.

Il paziente ospedalizzato potrebbe essere gestito anche con un’associazione precostituita di insulina basale e agonista del recettore del GLP1 praticabile, tra l’altro, anche con un’unica mono-somministrazione giornaliera dei due farmaci con la possibilità di ottenere un buon controllo sia della glicemia a digiuno che di quella post-prandiale, minimizzando il rischio di ipoglicemia. Tra l’altro, gli analoghi del GLP1 potrebbero offrire un teorico vantaggio dato il loro effetto anti-infiammatorio [19–21], anche se non esistono evidenze dirette nello specifico. Inoltre, poiché è stato osservato che alcuni virus della famiglia Coronavirus potrebbero doversi legare al recettore DPP4 (espresso anche nel sistema respiratorio) per entrare nelle cellule polmonari, è stato recentemente suggerito, sulla scorta di dati pre-clinici, che gli inibitori della DPP4 (aloglitin, linagliptin, saxagliptin, sitagliptin) potrebbero svolgere un effetto protettivo nei confronto dell’aggressione cellulare da parte del coronavirus [22]. Ovviamente, questa ipotesi rimane in attesa di verifica sul campo.

Quale che sia la terapia anti-iperglicemizzante in atto, gli obiettivi terapeutici sono quelli forniti delle più recenti linee guida internazionali per la gestione della persona con diabete ospedalizzata per patologie intercorrenti e cioè valori di glicemia compresi tra 140 e 180 mg/dl nel corso delle 24 ore, con la possibilità di perseguire target glicemici meno stringenti nei pazienti critici [23]. In questi ultimi dovrebbe comunque essere sempre considerato il rischio di cheto-acidosi diabetica, così come nel paziente in terapia nutrizionale parenterale va sempre valutato il rischio di una sindrome iper-osmolare. In caso di terapia insulinica endovenosa è necessario un attento monitoraggio della ionemia e, in particolare, della potassiemia in quanto l’insulina, facilitando il passaggio del potassio nel comparto intracellulare, può precipitare una condizione di ipokaliemia.

In tutti questi pazienti il monitoraggio glicemico è ovviamente fondamentale e utile può rivelarsi l’impiego dei sistemi di monitoraggio continuo della glicemia (CGM). Non solo questi sistemi permettono un controllo in continuo ma offrono anche il vantaggio di poter essere gestiti da remoto consentendo al personale sanitario di poter visionare i valori glicemici senza doversi recare al letto del paziente e di intervenire alla bisogna.

Infine, è utile ricordare che, al di là della persona con diabete noto ricoverata per infezione da COVID-19 in ambito ospedaliero, frequenti possono essere i riscontri di iperglicemia non nota probabilmente svelati dalla condizione di stress acuto e quelli conseguenti all’uso, sempre più diffuso e sempre più precoce per il trattamento di questi soggetti, di steroidi.

Oltre al trattamento anti-iperglicemizzante, la persona con diabete tende ad assumere molti altri farmaci per condizioni frequentemente associate al diabete che potrebbero richiedere una particolare riflessione. I soggetti con diabete tipo 2 sono spesso ipertesi e l’impiego, a fine nefro-protettivo, di ACE inibitori e sartani è molto comune. Soprattutto per gli ACE-inibitori è stato suggerito un possibile effetto di suscettibilità per la patologia polmonare in pazienti con infezione da COVID-19 [24]. Peraltro, in assenza di chiari dati clinici, l’Agenzia Italiana del Farmaco (AIFA) e la Società Italiana dell’Ipertensione Arteriosa (SIIA) sconsigliano al momento la sospensione di tali farmaci, fatti salvi i soggetti con sintomi e/o sepsi severa che vanno valutati caso per caso [25, 26].

## Diabete e nuovi test per la diagnosi di infezione da COVID-19

Gli studi condotti nel corso delle precedenti epidemie di SARS e MERS hanno evidenziato che i pazienti con infezione da coronavirus sviluppano una potente risposta immune di tipo umorale con comparsa di IgG, IgM e IgA specifiche. Le IgM raggiungono il picco nel siero durante la fase di infezione per poi diminuire e dare luogo a una crescita del titolo di IgG durante la fase di convalescenza. Queste ultime rappresentano la principale risposta neutralizzante contro il COVID-19, tale da aver suggerito il trattamento di pazienti critici con il plasma di pazienti convalescenti [27].

Al di là del possibile utilizzo a scopo terapeutico degli anticorpi anti COVID-19, essi sono anche un marcatore di esposizione al virus (IgM) e superamento (IgG) della fase acuta di contatto, anche se ancora dibattuta è la loro cinetica, così come ancora da definire in modo certo è la specificità, sensibilità e affidabilità dei test sierologici oggi disponibili. In ogni caso, l’atteggiamento più recente è quello di ricercare questi anticorpi nelle categorie a maggior rischio di contatto (ad esempio gli operatori sanitari), così come le categorie che maggiormente potrebbero avere conseguenze. Tra queste, le persone con diabete dovrebbero essere sottoposte a screening sierologico al fine di individuare i soggetti non ancora esposti al virus sui quali insistere con misure profilattiche aggiuntive [28].

## Conclusioni

L’epidemia di COVID-19 rappresenta un ulteriore motivo di preoccupazione per molte persone con diabete e una sfida al sistema sanitario. Apparentemente, la presenza della malattia diabetica non aumenta il rischio di infezione ma ne può condizionare un’evoluzione meno favorevole. L’ottimizzazione del controllo glicemico e l’adozione delle misure di prevenzione e contenimento possono svolgere un ruolo importante per minimizzare le conseguenze. In ogni caso, le persone con diabete devono essere considerate come un gruppo particolarmente vulnerabile per il quale specifiche strategie devono (e in buona pare sono state) essere poste in atto incluso un ampio screening sierologico per poter intervenire prontamente sia in termini di trattamento precoce che di misure di contenimento.

## Materiale elettronico supplementare

I link al materiale elettronico supplementare sono elencati qui sotto.

## Consenso informato

Lo studio presentato in questo articolo non ha richiesto sperimentazione umana.

## Studi sugli animali

Gli autori di questo articolo non hanno eseguito studi sugli animali.
