# Supplementary material for: Guida pratica alla prevenzione e gestione dell’infezione da COVID-19 nelle persone con diabete
Source: L'Endocrinologo. 2020 Oct 23;21(4):241–5. [Article in Italian] doi: 10.1007/s40619-020-00767-3 (PMC7582423; doi:10.1007/s40619-020-00767-3)
Supplement: Supplementary file 1 [file 40619_2020_767_MOESM1_ESM.docx]

**Scheda valutazione**

(4 quesiti a risposta multipla con 3 risposte ciascuno, di cui una sola esatta)

**1) Il rischio di infezione da Sars-CoV2 in una persona con diabete è?**

1. Inferiore a quello della popolazione generale
2. Uguale a quello della popolazione generale
3. Maggiore di quello della popolazione generale

**2) Nel caso si sospetti COVID-19 in un diabetico tipo 2 in terapia con SGLT-2 inibitori gestibile a domicilio è consigliabile**

1. Sospendere SGLT-2 ed intraprendere terapia con insulina basale
2. Monitorare chetonemia/chetonuria
3. Consigliare di diminuire l’introito calorico

**3) Nel diabetico tipo 2 ospedalizzato per COVID-19**

1. L’insulina in infusione endovenosa rappresenta la terapia d’elezione
2. Occorre monitorare la ionemia poiché l’insulina endovena espone al rischio di iperpotassiemia
3. Un buon controllo glicemico parte dalla dieta

**4) Se un paziente con diabete non ha potuto eseguire il rinnovo della patente nel mese di Maggio 2020 causa COVID-19**

1. Sarà sufficiente ripetere il fundus oculi per poter guidare
2. Non potrà guidare se l’ultima glicata era superiore a 75 mmol/l
3. la patente sarà in ogni caso valida fino alla fine di agosto
