# Supplementary material for: Guida pratica alla prevenzione e gestione dell’infezione da COVID-19 nelle persone con diabete
Source: L'Endocrinologo. 2020 Oct 23;21(4):241–5. [Article in Italian] doi: 10.1007/s40619-020-00767-3 (PMC7582423; doi:10.1007/s40619-020-00767-3)
Supplement: Supplementary file 2 [file 40619_2020_767_MOESM2_ESM.docx]

**Riposte**

( 4 quesiti a risposta multipla con 3 risposte ciascuno, di cui una sola esatta)

**1) Il rischio di infezione da Sars-CoV2 in una persona con diabete è?**

b) Uguale a quello della popolazione generale

**2) Nel caso si sospetti COVID-19 in un diabetico tipo 2 in terapia con SGLT-2 inibitori gestibile a domicilio è consigliabile**

b) Monitorare chetonemia/chetonuria

**3) Nel diabetico tipo 2 ospedalizzato per COVID-19**

c) Un buon controllo glicemico parte dalla dieta

**4) Se un paziente con diabete non ha potuto eseguire il rinnovo della patente nel mese di Maggio 2020 causa COVID-19**

c) la patente sarà in ogni caso valida fino alla fine di agosto
